# Head biomechanics of video recorded falls involving children in a childcare setting

**DOI:** 10.1038/s41598-022-12489-7

**Published:** 2022-05-21

**Authors:** Gina Bertocci, Craig Smalley, Nathan Brown, Raymond Dsouza, Bret Hilt, Angela Thompson, Karen Bertocci, Keyonna McKinsey, Danielle Cory, Mary Clyde Pierce

**Affiliations:** 1 Department of Bioengineering, University of Louisville, Louisville, KY USA; 2 Engineering Fundamentals Department, University of Louisville, Louisville, KY USA; 3 Feinberg School of Medicine, Northwestern University, Chicago, IL USA; 4 Division of Emergency Medicine, Ann & Robert H. Lurie Children’s Hospital, Chicago, IL USA

**Keywords:** Biomedical engineering, Trauma

## Abstract

The objective of this study was to characterize head biomechanics of video-recorded falls involving young children in a licensed childcare setting. Children 12 to < 36 months of age were observed using video monitoring during daily activities in a childcare setting (in classrooms and outdoor playground) to capture fall events. Sensors (SIM G) incorporated into headbands worn by the children were used to obtain head accelerations and velocities during falls. The SIM G device was activated when linear acceleration was ≥ 12 g. 174 video-recorded falls activated the SIM G device; these falls involved 31 children (mean age = 21.6 months ± 5.6 SD). Fall heights ranged from 0.1 to 1.2 m. Across falls, max linear head acceleration was 50.2 g, max rotational head acceleration was 5388 rad/s^2^, max linear head velocity was 3.8 m/s and max rotational head velocity was 21.6 rad/s. Falls with head impact had significantly higher biomechanical measures. There was no correlation between head acceleration and fall height. No serious injuries resulted from falls—only 1 child had a minor injury. In conclusion, wearable sensors enabled characterization of head biomechanics during video-recorded falls involving young children in a childcare setting. Falls in this setting did not result in serious injury.

## Introduction

Falls were the leading cause of non-fatal injuries in children 0–4 years of age in 2019 in the United States, accounting for 691,543 emergency department (ED) visits; this represented 45% of all injury-related ED visits in this age group^1^. Among children < 1 year of age the proportion of injury-related ED visits due to falls was even higher at 58%^1^. Immature motor skills and developing functional ambulatory skills during this period of life make infants and young children more susceptible to falls and fall-related injuries.

Injuries resulting from short distance falls involving young children have been the focus of numerous epidemiological studies^2,3^, retrospective and prospective clinical studies^4–8^, and biomechanical studies using surrogates^9–16^ and computational models^17–26^ of the body or head in simulated falls. Since falls are also the most common falsely provided history in child abuse^27^, many studies have sought to determine whether short distance falls can lead to serious or fatal injuries in children, while others have sought to identify types of injuries resulting from falls and/or fall types and other factors (e.g. child age, height) that influence injury risk in falls^4,7,28^. Most studies have concluded that short distance falls rarely lead to fatality^2,4–6,29^, with a small proportion of falls (2–6%) resulting in serious head injuries^5,6,8^. Unfortunately, most of these studies employed a retrospective design and/or lacked reliable witnesses capable of verifying or corroborating fall histories and associated fall details.

Findings from studies that have occurred in a hospital setting^30–33^, were video-recorded^3^ or corroborated by a second person other than a caregiver^29,34^ provide a more reliable assessment of injuries resulting from falls involving children. Although the definition of *short distance fall* was not standardized, when combining falls from these studies, 12 fractures, 3 sub-dural hemorrhages (SDH) and 2 fatal brain injuries occurred across 807 falls^29–34^. One fatality occurred when a child pushed another child into the fall victim who then fell rearward onto cobblestone pavement^29^. The other fatality occurred when a child straddling an elevated rail of play structure lost her balance, rotated laterally, and fell head-first onto carpet over concrete^3^. It is likely that initial velocity, fall dynamics (i.e., rotational motion), impact surface and region of head impact played an important role in these fatal outcomes. An additional study of children (< 4 years of age) presenting to the ED with a household fall history incorporated in-depth caregiver interviews and fall scene investigations, along with rigorous criteria to rule out abusive injuries from the dataset^8^. Falls in this study led to 2 small SDHs and 17 fractures in 79 falls; none of these children had multiple or fatal injuries. This study further demonstrated that fall details were important to injury outcomes.

Biomechanical studies investigating injury risk in falls typically rely upon instrumented surrogates and computer modeling to represent a specific pediatric population in fall experiments and simulations. Surrogate studies have evaluated the influence of fall height, impact surface and initial conditions on the likelihood of head injury^9–13,15,16,35,36^. Findings from these studies vary, with some indicating a low likelihood of head injury in falls from 0.3 to 0.7 m onto carpet^9,15,16^, one indicating a moderate risk of concussion in falls from 0.3 to 1.5 m onto concrete^13^, and another indicating an uncertain head injury risk in 1.5 m falls onto carpet and concrete^35^. Differences across studies are likely due to varying surrogate biofidelity and design, along with differences in child age represented by surrogates, initial position, fall dynamics^16^ and anatomic location of impact^15^. Computational modeling has been used to investigate the effects of fall characteristics, initial conditions, and child characteristics on head injury risk in bed falls^17,18^, as well as to evaluate the sufficiency of playground standards in protecting against head injury in children and assessing the influence of playground surface stiffness and region of impact on head injury risk in falls^19,20^. Additional computational modeling studies using finite element models of the infant skull and brain have also been conducted to predict skull fracture risk associated with head impact and falls^21–26^.

The lack of agreement across biomechanical studies, in combination with a relatively small number of reliably witnessed falls with limited fall details highlights the need for a more reliable means of characterizing falls involving young children while simultaneously capturing biomechanical measures. The objective of our study was to directly measure head biomechanics of video-recorded falls in a childcare setting using wearable sensors and to characterize the details of these falls. Additionally, we sought to describe injury outcomes associated with these falls and to determine the relationship between head injuries and biomechanical measures.

## Methods

### Study design

This study used a prospective, cross-sectional observational design. All study procedures were performed in accordance with the approved University of Louisville Institutional Review Board protocol (IRB #16.1030) and relevant guidelines/regulations. Legal guardians provided informed consent for all subjects.

### Inclusion criteria

Children 12 to < 36 months of age assigned to one of four childcare center classrooms. Children with bone disorders, bleeding disorders or neurological disorders were excluded from the study.

### Definitions

*Fall*  - an event causing an uncontrolled transition in a child’s center of mass (COM) from a higher elevation to a lower elevation. Only falls meeting this definition were included in the analysis.

*Fall height* was described using two methods: (1) change in child’s head COM, where head COM was defined as the mid-point of the child’s head height based on subject-specific anthropometrics, and (2) change in support surface height. For example, a fall from standing to the ground would be classified as having *no change in support surface*, while a fall to the ground from standing on a chair would be classified as a *fall from a higher surface*, and a fall up the stairs would be categorized as a *fall to a higher surface*. These two methods were used to determine fall height since *change in support surface height* is pertinent when describing the fall environment and *change in head COM* is relevant to head kinematics and head injury risk.

### Procedure

Fall events occurring during routine daily activities in 4 classrooms and an outdoor playground at a licensed childcare center were captured using video monitoring. Three digital video cameras (Lorex Technology, Markham, Canada) operating at 30 frames/s and 1080p resolution were positioned to monitor each space (15 cameras total); camera feeds were transmitted to a network video recorder (NVR; Lorex Technology, Markham Canada). Instrumented elastic headbands incorporating a wearable sensor [SIM G (Smart Impact Monitor); Triax Technologies, Norwalk CT], consisting of a triaxial accelerometer (780 Hz cut-off) and triaxial gyroscope (250 Hz cut-off), were snugly fit to each child’s head to collect biomechanical measures (linear and rotational head acceleration and rotational head velocity) during falls. Small (43 cm), medium (47 cm) and large (51 cm) sized headbands were specially fabricated (Triax Technologies, Norwalk CT) to fit head circumferences ranging from 5th percentile 1-year-old (43.4 cm) through 95th percentile 3-year-old children (51.3 cm). The SIM G device was activated when linear accelerations were ≥ 12 g (Triax pre-set threshold), in which case the device stored 10 ms of data before and 52 ms after the triggering event. SIM G data collected at 1000 Hz was transmitted over a 900 MHz radio frequency to a wi-fi connected aggregating receiver (Sky-i; Triax Technologies, Norwalk CT) and was automatically uploaded and stored in a cloud-based database. The receiver was located to assure SIM G devices were within its 138 m receiving range. NVR time was synchronized with the Sky-i receiver time prior to each data collection session.

Furniture, fixtures, and playground equipment heights were measured to document the environment. Additionally, coefficient of restitution (COR) was measured for potential impact surfaces using a resiliency tester (IDM Instruments, San Sebastian ESP; model F0020). Anthropometric measures were obtained for each subject at the beginning of the study and at 6-month intervals.

Video monitoring, in conjunction with researcher observation, was conducted 2–3 times/week for a 1.5–2-h period for 7 months. Video monitoring/observations alternated between two classrooms and the playground one week, followed by the opposite two classrooms and the playground the following week in an effort to evenly distribute monitoring hours. One researcher was located in each of the video monitored spaces to reapply instrumented headbands when they were removed by children. A unique SIM G ID number corresponding to the device assigned to each subject was recorded at the beginning of each observation period.

### Post-processing of video captured falls and SIM G data

Video footage from each camera was stored on an NVR drive during observation periods and was subsequently reviewed to identify fall events. Once a fall event was identified, time stamped video footage from all cameras in the monitored space was clipped to include 5 s pre-fall and 5 s post-fall; clipped files were saved and stored for analysis. SIM G data was reviewed to determine whether activation occurred during each fall based on fall time and subject ID obtained from video footage. In SIM G activated falls, peak linear and rotational head acceleration and peak rotational head velocity were extracted from recorded data. Additionally, peak change in linear head velocity was determined from integration of linear head acceleration. Impact duration was determined from linear acceleration time history. (Impact duration was defined as T_f_ − T_i_, where T_i_ is the time before the peak where the linear acceleration value is 10% of the peak and T_f_ is the time after the peak where linear acceleration is 10% of the peak.) Through review of video footage, each fall was classified by fall type as *no change in support surface height*, *fall from a higher surface,* or *fall to a higher surface*, and it was determined whether head impact occurred (*yes/no*) in the fall. Initial conditions, fall dynamics, and impact surface were also determined for each fall. Additionally, it was documented whether an injury occurred during the fall and if so, the injury was described. Whether or not first aid was required, and/or medical care was sought was recorded for each fall. In cases where incident reports were filed by childcare center staff, they were collected and reviewed for additional details regarding injuries, first aid and/or medical care.

### Data analysis

Data were evaluated for normality (Shapiro–Wilk test), homogeneity of variances (Levene’s test) and homogeneity of regression slopes (where appropriate); parametric or nonparametric statistical tests were conducted as appropriate. Independent variables (i.e., COR, fall type, child mass, head impact) were evaluated to identify covariates. Correlational tests (Pearson correlation coefficient for parametric data or Spearman rank order correlation coefficient for non-parametric data) identified COR as the only covariate. Independent ANCOVAs (where assumptions were met; change in linear velocity) or the non-parametric equivalent, Quade’s test (where assumptions were not met; linear and rotational acceleration, rotational velocity), were performed to determine if there were differences in biomechanical measures based on head impact and fall type while controlling for COR. For Quade’s test, each dependent variable (i.e., biomechanical measures—linear and rotational acceleration, rotational velocity) and the covariate (i.e., COR) were ranked, regression was performed using the ranks of the dependent variable (i.e., biomechanical measures) on the ranks of the covariate (i.e., COR) to obtain residuals, and analysis of variance was performed using the residuals as the dependent variable and the grouping variable (i.e., fall type or head impact) as the factor.

Relationships between biomechanical measures were investigated using partial correlations while controlling for independent factors (e.g., fall type, head impact). Bivariate correlations were performed to determine whether there were relationships between biomechanical measures (i.e., head accelerations) and fall height (independent variable), while partial correlations were used to determine the relationship between biomechanical measures (i.e., head accelerations) and fall height when controlling for various factors (i.e., fall type-*from a higher surface/no change in support surface height/to a higher surface*, head impact-*yes/no*, bracing-*yes/no*, playground rubber mulch impact surface-*yes/no*). The distribution of fall characteristics, including initial condition, fall dynamics and impact surface based on fall type (*from a higher surface/no change in support surface height/to a higher surface*) and head impact (*yes/no*) were also described. Descriptive statistics were determined for biomechanical measures. All analyses were performed using SPSS® Statistics Ver. 26 (IBM, 2020). P-values were considered statistically significant at p < 0.05.

### SIM G verification

To verify the performance of the SIM G sensor when incorporated into our specially sized headbands for young children, we compared measurements from CRABI-12 surrogate tri-axial head accelerometers and angular rate sensors (reference sensors; located at the head COM) to SIM G measurements in feet-first falls (n = 5). The surrogate was suspended 23 cm above a linoleum over wood floor and then released to simulate a feet-first fall as described in Thompson, et al.^37^. Findings from these experiments demonstrated: (1) a strong correlation between reference measurements and SIM G measurements, (2) no significant difference between reference measurements and the SIM G measurements, (3) a percentage difference ≤ 1.4% for all SIM G measurements, and (4) SIM G 95% confidence intervals (CI) that fell within the 95% CIs of the reference sensors, with the exception of the lower bound of rotational velocity which was 0.1 rad/s outside of the CRABI-12 95% CI (Table 1). Thus, for this impact severity and fall condition, the SIM G sensors demonstrated acceptable equivalence with the CRABI-12 reference sensors.

**Table 1:** Comparison of SIM g to CRABI-12 surrogate head kinematics in feet-first falls (n = 5).

	Linear acceleration	Rotational acceleration	Rotational velocity
Mean peak CRABI-12	30.9 g (± 3.2)	2617 rad/s^2^ (± 560)	13.7 rad/s (± 2.6)
Mean peak SIM G	31.0 g (± 1.4)	2597 rad/s^2^ (± 470)	13.5 rad/s (± 2.5)
Mean % difference	− 0.2%	0.8%	1.4%
T test p-value	0.978	0.953	0.907
R^2^ Slope	0.99 (p = < 0.001) y = 1.14x	0.92 (p = 0.01) y = 0.98x	0.92 (p = 0.01) y = 0.98x
95% CI CRABI-12	28.2–33.8	2127–3107	11.4–16.0
95% CI SIM G	29.8–32.2	2184–3010	11.3–15.8

## Results

### Demographics

35 children (mean age at enrollment: 19.4 months ± 5.8 SD) were enrolled in the study. In 269 observation hours (sum of hours in individual rooms and on playground) equating to 1050 subject-hours, SIM G devices were activated (≥ 12 g threshold) in 174 video-recorded falls (17% of falls with subjects wearing SIM G; Table 2); these falls involved 31 children who had a mean age of 21.6 months (± 5.6 SD) and a mean mass of 12.4 kg (± 1.4 SD) with anthropometrics as indicated in Supplementary Table S1. The majority of falls (79.3%) involved males.

**Table 2:** Summary of video-recorded falls.

No. of video-recorded falls with subjects wearing SIM G	1021
No. of video-recorded falls where SIM G was activated (head acceleration ≥ 12 g)^a^	174

(269 observation hours;  (1050 subject-hours).

^a^847 falls < 12 g.

### SIM G activation rates

SIM G activation occurred at a rate of 0.2 activations/subject-hour of observation (i.e., 0.2 activations occurred while observing and videorecording 1 subject for 1 h); a higher rate of activation (0.4 activations/subject-hour) occurred on the playground. The SIM G was activated in 27% (n = 47 of 174 with SIM G worn) of falls from a higher surface and in 15% (n = 122 of 808 with SIM G worn) of falls with no change in support surface. Results that follow are limited to the 174 falls where SIM G activation occurred.

### Fall characteristics

**Figure 1:**
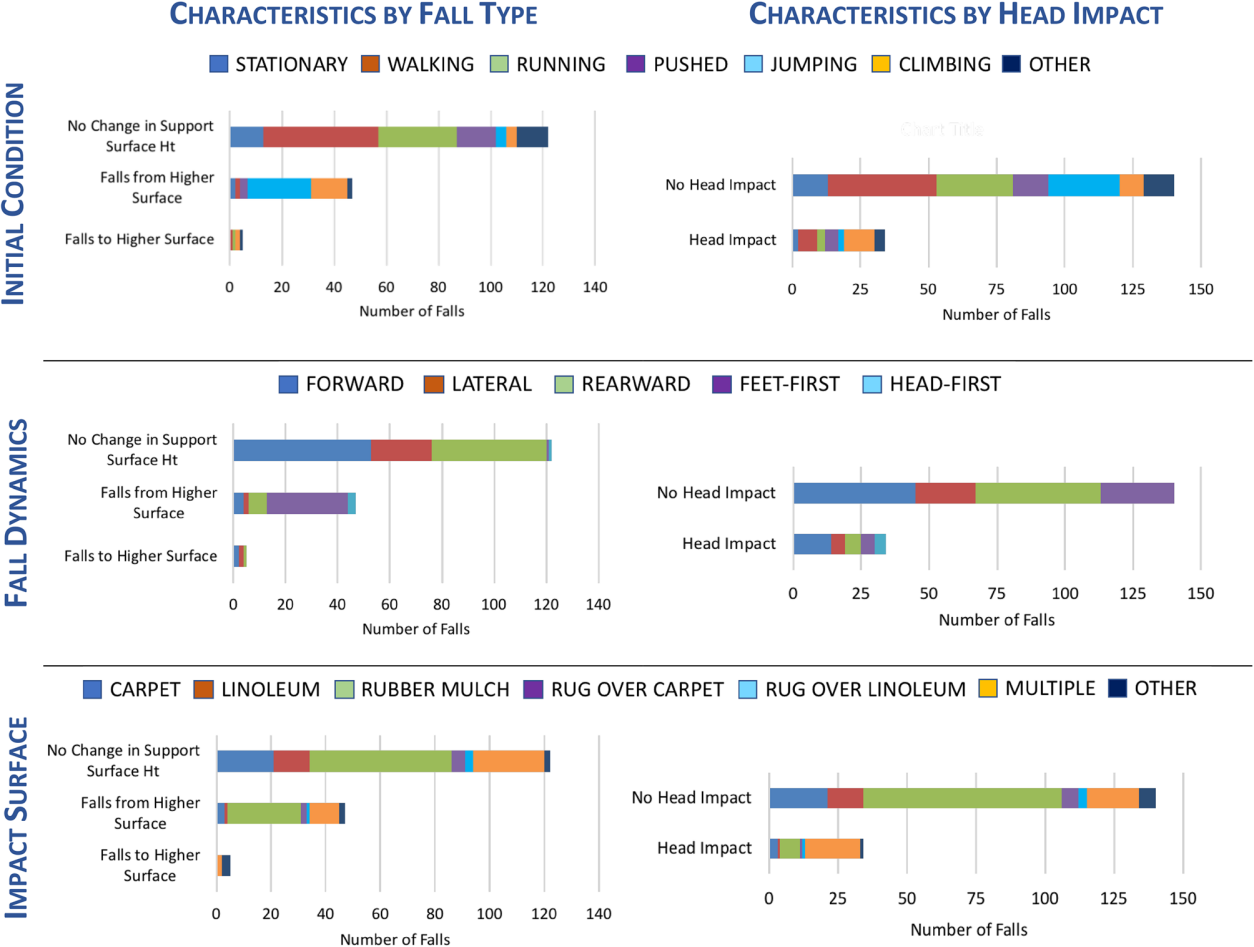
Initial condition, fall dynamics, and impact surface based on fall type and head impact (n = 174).

**Figure 2:**
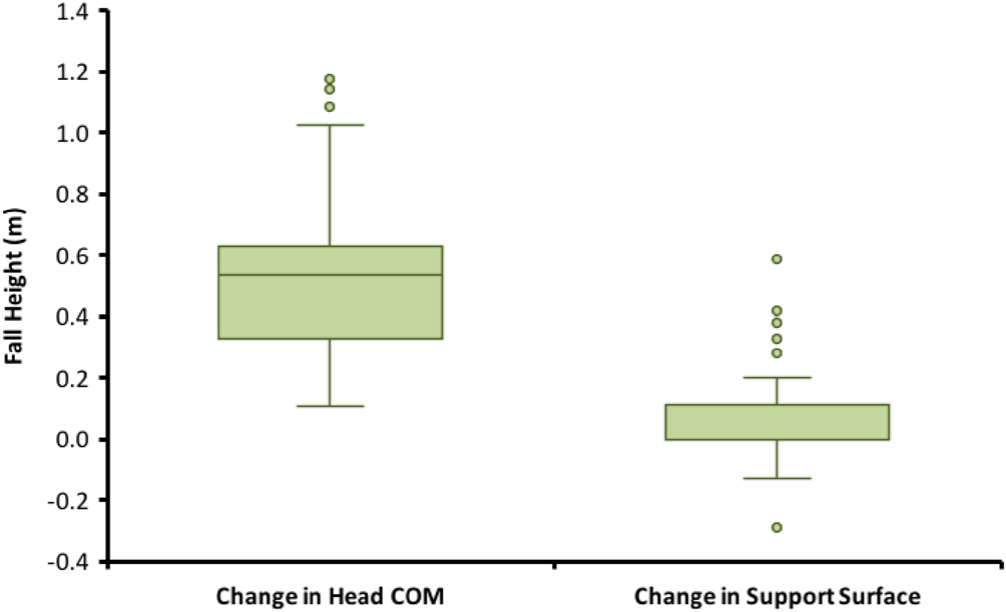
Distribution of fall heights defined as change in head COM and change in support surface height (n = 174). 25th and 75th percentiles are represented by the lower and upper bounds of box, respectively. Line within the box represents median value, and the whiskers represent 1.5 times the interquartile range (IQR; IQR = 75th percentile value minus 25th percentile value). Note: median and 25th percentile values are coincident (0.0) for change in support surface height.

Falls were characterized by initial condition, primary fall dynamic, and impact surface based on fall type and head impact (*yes/no*) (Fig. 1). For falls with SIM G activation, 55% (n = 96) of falls occurred on the playground and 27% (n = 47) of falls were from a higher surface, most of which were feet-first falls (55%; n = 26 of 47) on the playground. The maximum change in support surface height was 0.59 m and the maximum change in head COM was 1.2 m (Fig. 2). In falls where there was no change in support surface height (70%; n = 122), most were either forward or rearward falls that occurred during walking. The majority of these falls (42.6%; n = 52) occurred on the playground. Overall, children impacted their head in only 19% (n = 34) of falls. Thus, most head accelerations were associated with indirect loading of the head. Climbing was the predominant initial condition (32%; n = 11) in falls where head impact occurred. Most falls with head impact involved forward fall dynamics (41%; n = 14).

### Head biomechanics

**Figure 3:**
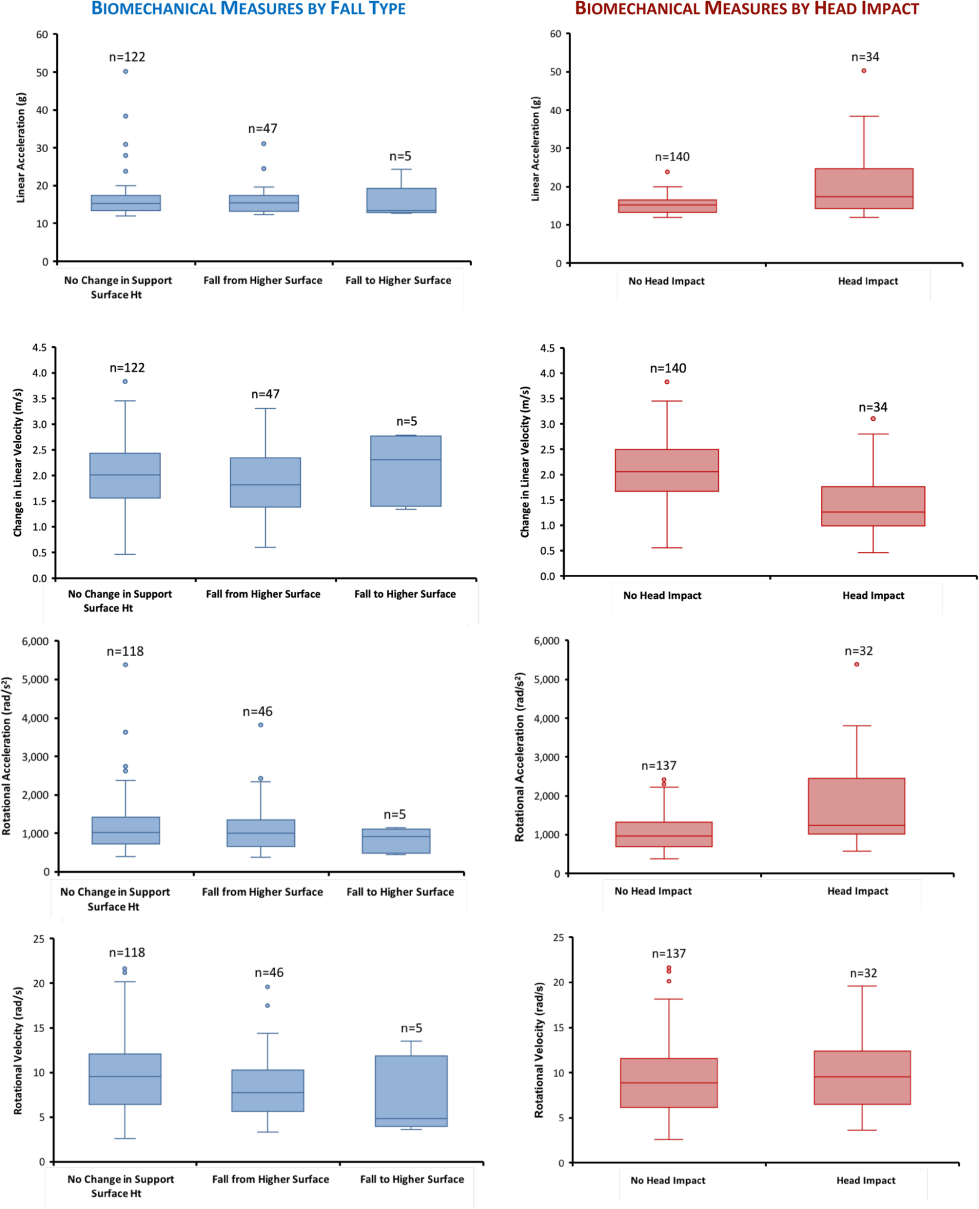
Box plots of peak linear head acceleration, change in linear head velocity, rotational head acceleration and rotational head velocity by fall type (left column) and head impact (yes/no) (right column). 25th and 75th percentiles are represented by the lower and upper bounds of the box, respectively. Horizontal line within the box represents median value, and the whiskers illustrate the minimum and maximum values unless there are outliers. When outliers are present, whiskers represent 1.5 times the interquartile range (IQR; IQR = 75th percentile value minus 25th percentile value). (Note: SIM G sensor did not record rotational acceleration/velocity for 5 falls.)

The maximum recorded linear acceleration across falls was 50.2 g, which occurred coincident with the maximum rotational head acceleration (5388 rad/s^2^) (Table 3). These accelerations occurred when a 17-month-old child tripped while walking, falling forward and directly impacting his head on the side of a wooden bookcase. (Detailed fall descriptions for three of the highest accelerations, along with associated acceleration time histories, are provided in Supplementary Table S2.) In general, head accelerations and velocities varied widely across falls (Table 3), with some biomechanical measures differing based upon fall type and head impact (Fig. 3).

**Table 3:** SIM G data for video-recorded falls (n = 174).

	Peak linear head acceleration (g)	Peak change in linear head velocity (m/s)	Peak rotational head acceleration (rad/s^2^)	Peak rotational head velocity (rad/s)	Impact duration (msec)
Median	15.3	1.9	1013	8.9	22.0
IQR^a^	4.0	0.9	675	5.6	8.0
Range	12.0–50.2	0.5–3.8	377–5388	2.6–21.6	6.0–34.0

Rotational acceleration/velocity was recorded for 169 falls—SIM G device did not record rotational acceleration/velocity for 5 falls.

^a^IQR, Interquartile Range = 75th percentile value–25th percentile value.

When comparing biomechanical measures based on fall type (Fig. 3), rotational head velocity was significantly greater in falls with no change in support surface height vs. falls from a higher surface (F_(1,162)_ = 5.13, p = 0.025) when controlling for impact surface COR. (COR values are provided in Supplementary Table S3). Linear head acceleration (F_(1,167)_ = 0.87, p = 0.35) and velocity (F_(1,167)_ = 1.56, p = 0.21), and rotational head acceleration (F_(1,162)_ = 0.01, p = 0.94) did not differ across fall types.

Whether or not head impact occurred influenced all biomechanical measures (Fig. 3) except rotational velocity (F_(1,162)_ = 1.54, p = 0.217). Linear head acceleration (F_(1,167)_ = 5.86, p = 0.017) and rotational head acceleration (F_(1,162)_ = 10.42, p = 0.002) were significantly greater in falls with head impact compared to those with no head impact, when controlling for impact surface COR. Conversely, change in linear head velocity (F_(1,167)_ = 25.48, p =  < 0.001) was significantly lower in falls with head impact.

**Figure 4:**
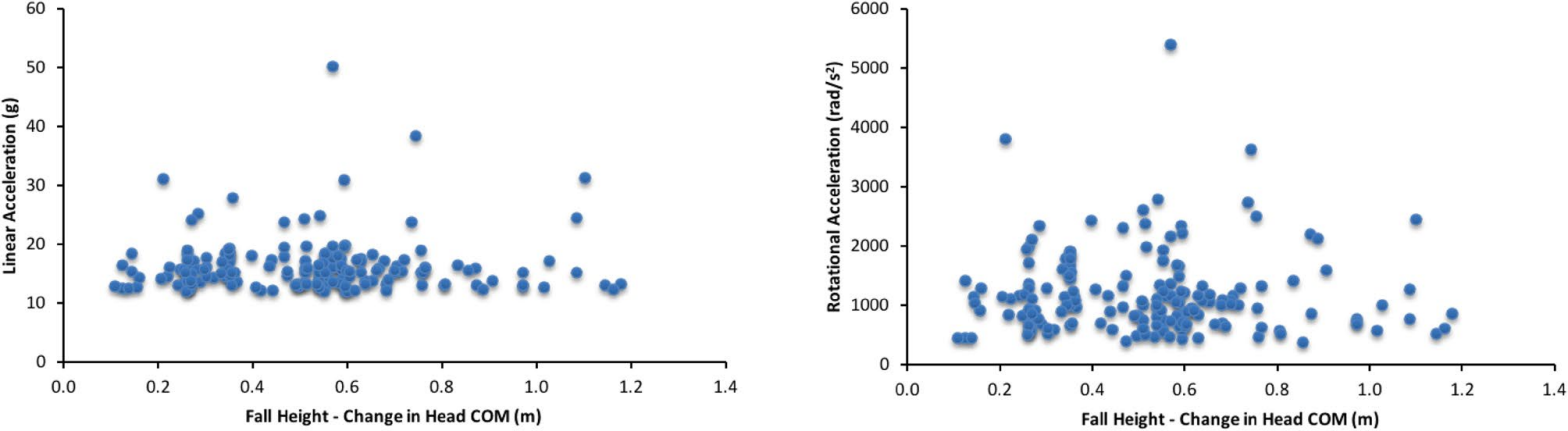
Linear head acceleration (n = 174) and rotational head acceleration (n = 169) vs. fall height (change in head COM).

For the range of fall heights in our study (0.1–1.2 m), neither linear head acceleration (r_s_ = − 0.004, p = 0.96) nor rotational head acceleration (r_s_ = − 0.056, p = 0.473) were related to fall height, where fall height was defined as the change in head COM (Fig. 4). The absence of a relationship between linear acceleration and fall height, and rotational acceleration and fall height persisted when independently controlling for fall type (r_s_ = 0.017, p = 0.83; r_s_ = 0.027, p = 0.733), head impact (r_s_ = − 0.034, p = 0.663; r_s_ = − 0.072, p = 0.360), playground rubber mulch impact surface (r_s_ = 0.041, p = 0.598; r_s_ = -0.014, p = 0.858) and whether children braced themselves during falls (r_s_ = 0.065, p = 0.405; r_s_ = 0.012, p = 0.881). When these same factors were controlled for in combination, no relationship between linear acceleration and fall height (r_s_ = 0.050, p = 0.525), or rotational acceleration and fall height (r_s_ = 0.007, p = 0.927) was found.

**Figure 5:**
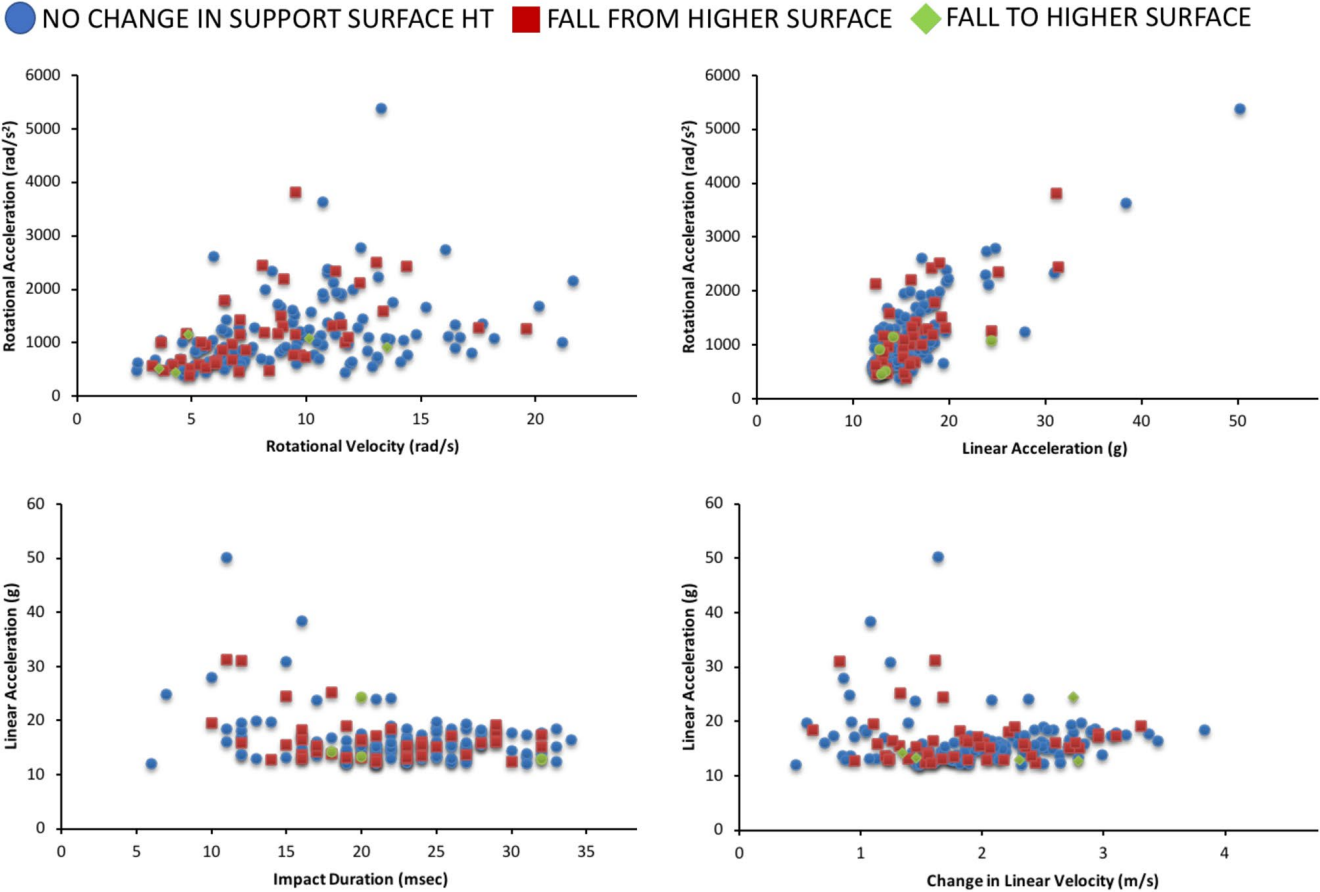
Relationships between biomechanical measures based on fall type (n = 174, except for rotational acceleration and velocity where n = 169).

**Figure 6:**
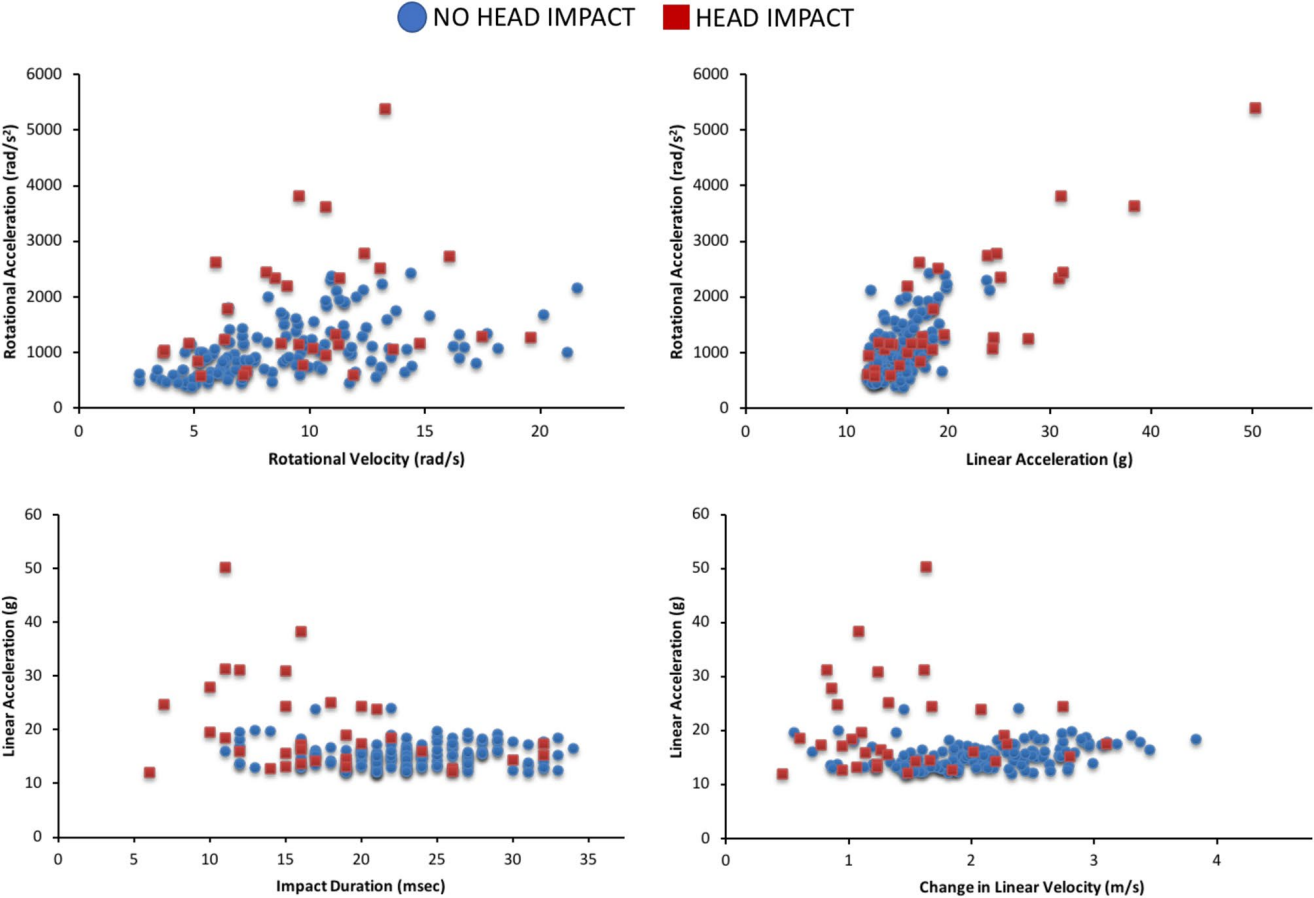
Relationships between biomechanical measures based on head impact (n = 174, except for rotational acceleration and velocity where n = 169).

Relationships between biomechanical measures were explored while controlling for fall type and head impact (Figs. 5 and 6). We found a strong relationship between rotational acceleration and linear acceleration when controlling for fall type (r = 0.80, p < 0.001; Fig. 5) and head impact (r = 0.80, p < 0.001; Fig. 6). Relationships between other biomechanical measures were either weak or did not exist.

### Injuries from falls

No serious injuries resulted from 174 falls with SIM G activation; medical care was not sought for any fall-related injuries. Only 1 child had a minor injury, a lip laceration, yielding a minor injury rate of 0.57%. In this fall, the child was attempting to step up onto the distal end (base) of the playground slide from a standing position—his foot slipped off the slide, causing him to fall forward onto his knee and hands as his face impacted the protruding hard plastic side rail of the slide. The child cried in response to the injury and was provided with first aid (ice pack) by the childcare center staff. No incident report was completed for this fall. This fall resulted in a 24.8 g linear head acceleration over a 7 ms impact duration and a 2781 rad/s^2^ rotational head acceleration.

Only one incident report was completed for our cohort of falls—it involved a childcare center staff member falling onto a child; there were no injuries in this fall. Given the absence of head injuries across falls, it was not possible to describe relationships between biomechanical measures and head injuries as intended.

## Discussion

To our knowledge, this is largest collection of video-recorded falls with simultaneous biomechanics measurement involving young children. Although falls occurred in a supervised childcare setting, they occurred while children were playing and engaged in daily activities, both indoors and outdoors on a playground. Reliably witnessed or video-recorded falls are critical to advancing our understanding of injuries that can result from short distance falls involving children. Across 174 video-recorded falls involving children in a childcare setting, wearable sensors measured a maximum linear head acceleration of 50 g and rotational head acceleration of 5388 rad/s^2^, both for the same fall. Consistent with previous reports of low mortality in short distance falls^2^, none of the falls in our cohort resulted in serious injury or fatality and only 1 fall led to a minor injury (lip laceration not requiring medical care). Recent studies employing a wearable array of head accelerometers in youth (9 to 14 years of age) football determined that a mean peak linear head acceleration of 62 g ± 29.7 g and a mean peak rotational head acceleration of 2609 ± 1591 rad/s^2^ were associated with concussions^38–40^. None of our measured linear head accelerations exceeded this concussion threshold, and only 1.1% (n = 2) of falls generated values within 1 standard deviation (32 g to 91 g) of this mean linear acceleration. However, 3.5% (n = 6) of falls in our study exceeded the mean peak rotational acceleration associated with concussions^38^ and 46% (n = 80) of our rotational accelerations fell within 1 standard deviation of the mean reported by Campolettano et al. (2020). (Other published concussion thresholds for high school/college athletes (102 ± 33 g and 4412 ± 2326 rad/s^2^) and professional football players (98 ± 28 g and 6432 ± 1813 rad/s^2^) are higher than those for youth football players^41,42^.) Campolettano et al. (2020) also proposed an aggregate measure based on the Generalized Acceleration Model for Brain Injury Threshold (GAMBIT)^43^ to account for the combined effects of both linear and rotational accelerations to improve prediction of concussion in children. Using Campolettano’s proposed GAMBIT concussion risk curve developed for children 9 to 14 years of age, only 1 fall in our cohort would have placed a child at a 25% risk of concussion; all others had less than a 25% risk of concussion. Since concussion thresholds tend to decrease with decreasing age^38–42^, thresholds for younger children such as in our study could be lower than those proposed by Campolettano. However, no children in our study had a concussion (operationalized as loss of consciousness or altered consciousness at the time of the fall or concussion-related symptoms such as vomiting or headaches post-fall).

While wearable sensors have previously been used to characterize head biomechanics in youth sports^40,44–52^, to our knowledge this is the first study capturing head biomechanics using these devices in pediatric falls. (Only one other study has used wearable sensors to investigate pediatric falls in a controlled laboratory setting but comprehensive biomechanics data was not reported^53^.) Prior to this study, head accelerations associated with falls involving infants and young children have been estimated by simulating falls using mechanical surrogates^9–16,36,54^. Although a direct comparison between our falls and surrogate simulated falls is difficult given the wide array of fall dynamics and impact surfaces, it appears that surrogates may over-estimate head accelerations in some falls. At similar fall heights, falls with head impact in our study generally resulted in lower head accelerations than those predicted using surrogates representing a similar age group^9,13,16,54^. For example, bed falls from 0.61 m using a CRABI-12 onto various surfaces led to mean peak linear head accelerations between 76 and 264 g^16^. Similarly, when using a Hybrid III—3 year old surrogate to investigate bed falls from 0.68 m onto various surfaces, Bertocci et al. reported mean peak linear head accelerations ranging from 110 to 220 g. These values exceeded all linear head accelerations measured in our study. Rotational accelerations reported for bed falls using the CRABI-12 and supine head first falls from 0.3 to 0.6 m using a surrogate representing an 18 month-old also greatly exceeded those measured in our study^13,16^. Differences between our findings and those from surrogate studies are likely due to differing fall dynamics (i.e. head-first impact vs. first impact to another body region), the ability of children to actively brace themselves during falls (thereby preventing or limiting head impact severity), or perhaps a surrogate head or neck that lacks biofidelity^15^. Interestingly, when compared to CRABI-12 surrogate rearward falls from standing onto carpet, playground foam and linoleum over wood, mean peak linear head accelerations (38–54 g) were within the range of values measured in our study, but the same fall onto wood or concrete generated values (120–132 g) in excess of our study^54^. Similarly, mean peak rotational head accelerations for these CRABI-12 falls onto carpet, playground foam and linoleum over wood (2700–4200 rad/s^2^) were comparable to our findings, but values for falls onto wood and concrete (6500–7800 rad/s^2^) exceeded those measured here. Falls in our study onto linoleum over concrete (n = 14) generated head accelerations ranging from 12 to 38 g and 450 to 3624 rad/s^2^—well below those reported by Thompson, et al. However, children braced themselves in 9 of these falls and direct head impact occurred in only 1 fall.

Across the range of fall heights (0.1 m to 1.2 m change in head COM) in our study, head accelerations did not increase with increasing fall height (Fig. 4). This may be due to not all falls involving direct head impact, maximum fall height being limited to 1.2 m, and children bracing themselves in many falls. However, even for falls with head impact, head accelerations did not increase with increasing fall height. Additionally, we found that falls from a higher surface were not associated with higher head accelerations or velocities. This is contrary to previous clinical studies suggesting falls from heights are more likely to be injurious, and thus would be associated with higher levels of head acceleration. In a case–control clinical study involving witnessed falls, Hughes et al. (2015) concluded that children < 4 years of age, and especially infants, were more likely to sustain a skull fracture or intracranial injury (ICI) in falls from > 0.6 m (2 ft) as measured from their head COM^28^. They further reported a 50% probability of skull fracture or ICI in falls from 1.54 m, with a mean fall height of 1.12 m for children who had simple skull fractures due to falls. In our study, 28.7% (n = 50) of falls were between 0.6 m and 1.2 m; none of these falls produced head injuries. Hughes et al. predicted a 15% probability of skull fracture or ICI at our maximum fall height of 1.2 m. Differences between our findings and those of Hughes et al. may be due to falls onto differing impact surfaces, estimated or caregiver-reported fall heights in the Hughes et al. study, differing fall dynamics, and/or differences as to whether direct head impact occurred. Other retrospective clinical studies suggested that fall height influenced injury severity^4–6^. Burrows et al. found that children were 2.9 times more likely to sustain a skull fracture or ICI when falling from a height vs. from standing^6^. Although fall heights were not reported by Burrows, they were described based on the object the child fell from; more children had abnormal head CTs when falling from the arms of an adult or falling from a window. Chaudhary et al. reported children (age 0–4 years) were 2.45 times more likely to have a severe injury when falling from a height between 1 m and 6 m compared to falling from < 1 m. In their study, children < 1 year of age were involved in more low-level falls yet had the majority of severe head injuries^4^. Mulligan et al. similarly reported more head injuries (e.g. skulls fractures, intracranial hemorrhage) when infants were dropped by caregivers, but fall heights were not reported^5^. Ibrahim et al. evaluated the influence of age and fall type (i.e., categorized range of fall heights) on head injury outcomes and found that although head injury patterns (i.e., type) differed by age and fall type, injury severity did not differ^7^. Differences between our study findings vs. previous clinical studies may be due to differing study design (prospective vs. retrospective), witnessed/video-recorded vs. caregiver reported falls, measured fall height vs. estimated fall height or using fall mechanism as a proxy for height, differing environments (supervised vs. unsupervised play, licensed childcare setting limiting height of climbing equipment and impact surfaces vs. home or other environments), and/or differences in the age of children (e.g., whether children < 1 year of age were included). Perhaps our injury outcomes would have differed if infants < 1 year of age were included, if fall heights exceeded 1.2 m, or if more falls occurred onto the linoleum over concrete surface.

Most of the falls from higher surfaces (n = 37 of 47 falls; 79%) in our study occurred on the playground, which had a rubber mulch surface; but falls from higher surfaces did not lead to higher head accelerations. Comparable rubber playground surfaces have been shown to reduce linear head acceleration in surrogate simulated falls^9,16^. Similar to clinical study findings described above, some surrogate and computer modeling studies representing a similar age group to those in our study have reported increasing head accelerations with increasing fall heights suggesting increased risk of head injury^11,13,15,17^. Our findings may differ from these surrogate studies due to a more compliant impact surface, as well as age-appropriate playground equipment having a limited maximum height, along with potential differences in fall dynamics and the ability of children to brace themselves during a fall. Additionally, the body region making initial contact with the impact surface and whether head impact occurred can also greatly influence head accelerations. In our study, only 34% (n = 16 of 47 falls) of falls from higher surfaces involved head impacts since most children effectively braced themselves during falls. Amongst these falls from a height that involved head impact, there were weak relationships between fall height and linear acceleration (r_s_ = − 0.39; p = 0.13) and fall height and rotational acceleration (r = − 0.46; p = 0.08). Thus, in this range of fall heights where head impact occurred, risk of head injury did not increase with increasing fall height. It is important to note however, that impact surface and fall dynamics varied across these falls—were they to remain constant, findings may differ. However, similar to our study findings, a surrogate study by Thompson et al. (2009) investigating feet-first falls did not identify a positive relationship between head accelerations and fall height for falls onto wood, linoleum over concrete, and carpet^54^. Additionally, Ibrahim et al. found no relationship between angular head acceleration and fall height for 0.6 m to 0.9 m fall heights onto carpet or for 0.3 m to 0.9 m fall heights onto concrete^13^.

Our falls involving direct head impact did not result in skull fractures, even at fall heights reported by others to cause skull fractures. In post-mortem human subject studies conducted by Weber (1984; 1985), infant cadaver (0–9 months of age) drops from 0.82 m (2.67 ft) with head impacts onto various surfaces produced skull fractures; all skulls (n = 15) were fractured in drops onto stone, carpet and linoleum, while only 1 of 10 skulls fractured in drops onto foam rubber and 4 of 25 skulls were fractured in drops onto a folded camel hair blanket^55,56^. Our falls from ≥ 0.82 m with direct head impact did not result in skull fractures or other injuries. Differences in our injury outcomes vs. post-mortem study findings could be due to differences in mechanical properties of the skull across age groups^57^, differing mechanical properties associated with post-mortem specimens vs. living human children, and/or the ability of children to brace themselves during falls. Van Ee et al. recreated cadaver drops conducted by Weber using a CRABI-6 surrogate to determine head accelerations associated with these falls^58^. On average, head accelerations ranged from 57 to 121 g and 4100–12,700 rad/s^2^ depending upon impact surface. Using injury probability models Van Ee predicted a 5% risk of skull fracture for a linear head acceleration of 50 g, which corresponds with our maximum recorded head acceleration in a direct head impact fall onto wood that resulted in no injury. However, when comparing our falls from ≥ 0.8 m with head impact, our head accelerations were much lower (15–24 g; 578–2510 rad/s^2^) than those measured by Van Ee using the CRABI-6; these falls were either onto playground mulch or rug over carpet laid on concrete. By comparison Van Ee reported peaks of 75 g and 7000 rad/s^2^ for falls onto a 2 cm thick foam mat and 57 g and 4100 rad/s^2^ for falls onto an 8 cm thick multiple folded camel hair blanket, which greatly exceed our findings. Differences may be attributable to differing fall dynamics, the presence of active muscle response in children and/or a lack of surrogate head/neck biofidelity. It is also likely that children fall differently than surrogates unless they are dropped by a caregiver or fall head-first from an elevated surface where there is unimpeded head-first contact. Additionally, compared to the rigid aluminum CRABI-6 head, the more compliant skull of a child^57^ likely serves to increase impact duration, thereby decreasing linear head acceleration.

Our study had the following limitations. It was conducted in a single licensed childcare center where children were supervised at an 8:1 child-to-staff ratio and the environment was designed to be “safe” (e.g., playground rubber mulch, limited height of playground equipment). Falls in other childcare centers, unlicensed childcare centers, or home environments may occur at a higher frequency, result in differing fall dynamics, or may lead to serious injuries with less supervision and a less safe environment. Another limitation is that falls with head accelerations < 12 g did not activate the SIM G sensor and thus, data were not recorded. Therefore, our cross-section of falls likely represents more severe falls occurring in the childcare center. Since we did not conduct skin assessments after falls, minor injuries such as bruising could have occurred. Although we did not follow fall victims beyond our observation time at the childcare center, we did observe children immediately after falls and in no case was there loss of consciousness, altered consciousness, or indications for medical attention. Moreover, we did not have any subsequent reports of required medical attention or reports of symptoms associated with concussions such as vomiting or headaches. Additionally, although we utilized specially sized headbands for our study population and assigned them to subjects based on head circumference to assure a snug fit, it is possible that headbands were not properly positioned or the SIM G moved relative to the head during falls, which could have introduced artifact or errors in recorded biomechanical measures. In an effort to limit such circumstances, on-site researchers and childcare staff re-positioned headbands when they were worn improperly, and data was reviewed to identify and remove falls with artifact from our dataset. Lastly, although we verified SIM G performance in feet-first falls using the CRABI-12 surrogate, accuracy of the SIM G may vary with differing fall dynamics or conditions as reported in other studies^59–64^. Additionally, coupling between the SIM G sensor and the surrogate head via the headband may differ from coupling between the SIM G and a child’s head given higher frictional properties of the surrogate head. Although forehead soft tissue indentation or erythema was often present upon removal of the headbands suggesting a snug fit, decreased coupling between a child’s head and the SIM G could lead to SIM G movement relative to the head potentially resulting in measurement inaccuracies during falls. However, as previously stated, through use of specially sized headbands for our study population we sought to minimize movement of the SIM G relative to the head.

## Conclusions

To our knowledge this is the first study to simultaneously capture video and head biomechanics of young children involved in falls in a licensed childcare center. This enabled us to characterize fall dynamics, fall height, impact surface, head biomechanics and injury outcomes for 174 falls. While children were involved in indoor and outdoor activities in a licensed childcare center, naturally occurring falls that met or exceeded the 12 g SIM G threshold (17% of falls) resulted in linear head accelerations ranging from 12 to 50 g and rotational head accelerations from 377 to 5388 rad/s^2^. No fatalities or serious injuries resulted from our cohort of falls, and only 1 child had a minor injury (laceration not requiring medical care); no children had multiple injuries.

## Supplementary Information

Supplementary Information.

## Data Availability

The dataset generated in this study is not publicly available since the data are part of on-going analysis and further manuscript development. Data will be made available upon request to the corresponding author once study manuscripts have been published.
