# Supplementary material for: Head biomechanics of video recorded falls involving children in a childcare setting
Source: Sci Rep. 2022 May 21;12:8617. doi: 10.1038/s41598-022-12489-7 (PMC9124183; doi:10.1038/s41598-022-12489-7)
Supplement: Supplementary file 1 — Supplementary Information. [file 41598_2022_12489_MOESM1_ESM.docx]

**HEAD BIOMECHANICS of VIDEO RECORDED FALLS INVOLVING CHILDREN in a CHILDCARE SETTING**

Gina Bertocci^1*^, Craig Smalley^1†^, Nathan Brown^1†^, Raymond Dsouza^1†^, Bret Hilt^1†^, Angela Thompson^2^, Karen Bertocci^1^, Keyonna McKinsey^1^, Danielle Cory^1^, Mary Clyde Pierce^3,4^

^1^ - Department of Bioengineering, University of Louisville, Louisville KY

^2^ - Engineering Fundamentals Department, University of Louisville, Louisville KY

^3^ - Feinberg School of Medicine, Northwestern University, Chicago IL

^4^ - Division of Emergency Medicine, Ann & Robert H. Lurie Children’s Hospital, Chicago IL

* - corresponding author

† - equivalent author contributions

**Supplementary Table S1. Anthropometrics of subjects in falls with** SIM™ G **data**

| **Age (month)** |  | **Weight (kg)** | **Height (cm)** | **Knee to Sole (cm)** | **Hip to Sole (cm)** | **Chest Depth (cm)** | **Shoulder Breadth (cm)** | **Hip Breadth (cm)** |
| --- | --- | --- | --- | --- | --- | --- | --- | --- |
|  | Median | 12.2 | 77.7 | 18.5 | 31.0 | 12.5 | 20.5 | 17.5 |
| 12-24^a^ | IQR^c^ | 0.9 | 3.2 | 1.5 | 1.0 | 1.3 | 1.0 | 0.8 |
|  | Range | 10.0-14.1 | 71.0-91.0 | 16.6-21.6 | 26.6-36.6 | 12.0-13.3 | 19.5-22.5 | 16.0-19.5 |
|  | Median | 14.0 | 91.0 | 21.6 | 36.8 | 13.0 | 22.3 | 18.5 |
| >24-36^b^ | IQR^c^ | 1.5 | 3.4 | 1.6 | 3.1 | 1.0 | 1.5 | 1.0 |
|  | Range | 12.0-16.6 | 83.0-95.9 | 19.1-22.1 | 31.6-40.1 | 12.0-14.0 | 19.0-24.5 | 17.5-19.5 |

| **Age (month)** |  | **Head Circumference (cm)** | **Head Height (cm)** |
| --- | --- | --- | --- |
|  | Median | 47.5 | 17.6 |
| 12-24^a^ | IQR^c^ | 1.5 | 0.2 |
|  | Range | 43.0-50.0 | 15.0-19.5 |
|  | Median | 50.00 | 18.6 |
| >24-36^b^ | IQR^c^ | 1.2 | 1.8 |
|  | Range | 47.7-52.5 | 17.4-21.5 |

^a^ - n=132 for 12-24 month age group

^b^ - n=42 for >24-36 month age group

^c^ - IQR, Interquartile Range = 75^th^ percentile value - 25^th^ percentile value

Note: Subject height was measured using a portable stadiometer (Hopkins Medical Products; Road Rod Portable Stadiometer) and weight was obtained using a scale (Health o Meter; Grow with Me 2 in 1 Baby to Toddler Scale). Hip and knee heights (from sole) were measured using the stadiometer. Head height was measured from chin elevation to the vertex of the head. Head circumference was measured using a flexible tape measure (Sammons Preston; Gulick Tape Measure), while shoulder breadth, hip breadth, and chest depth were measured using a caliper (Martin; 12-1230 Breadth Caliper).

**Supplementary Table S2. Falls associated with highest head accelerations - Fall descriptions and head acceleration time histories**

| **Fall 1** - The highest head accelerations (50.2 g; 5388 rad/sec^2^; 11 msec impact duration; Figure S2-1) were generated when a child was walking forward, tripped and fell forward impacting their head directly onto the side of a wooden bookcase. The child did not attempt to brace himself during the fall. Head impact occurred approximately 30 cm (12”) above the floor. Immediately following the fall, the child remained in a prone position on the floor crying. A childcare center staff picked up the child and consoled him. No first aid was required, no medical attention was sought, and no injuries were reported. The same child was observed standing and engaged in activities approximately 40 sec after this fall.   \| \| 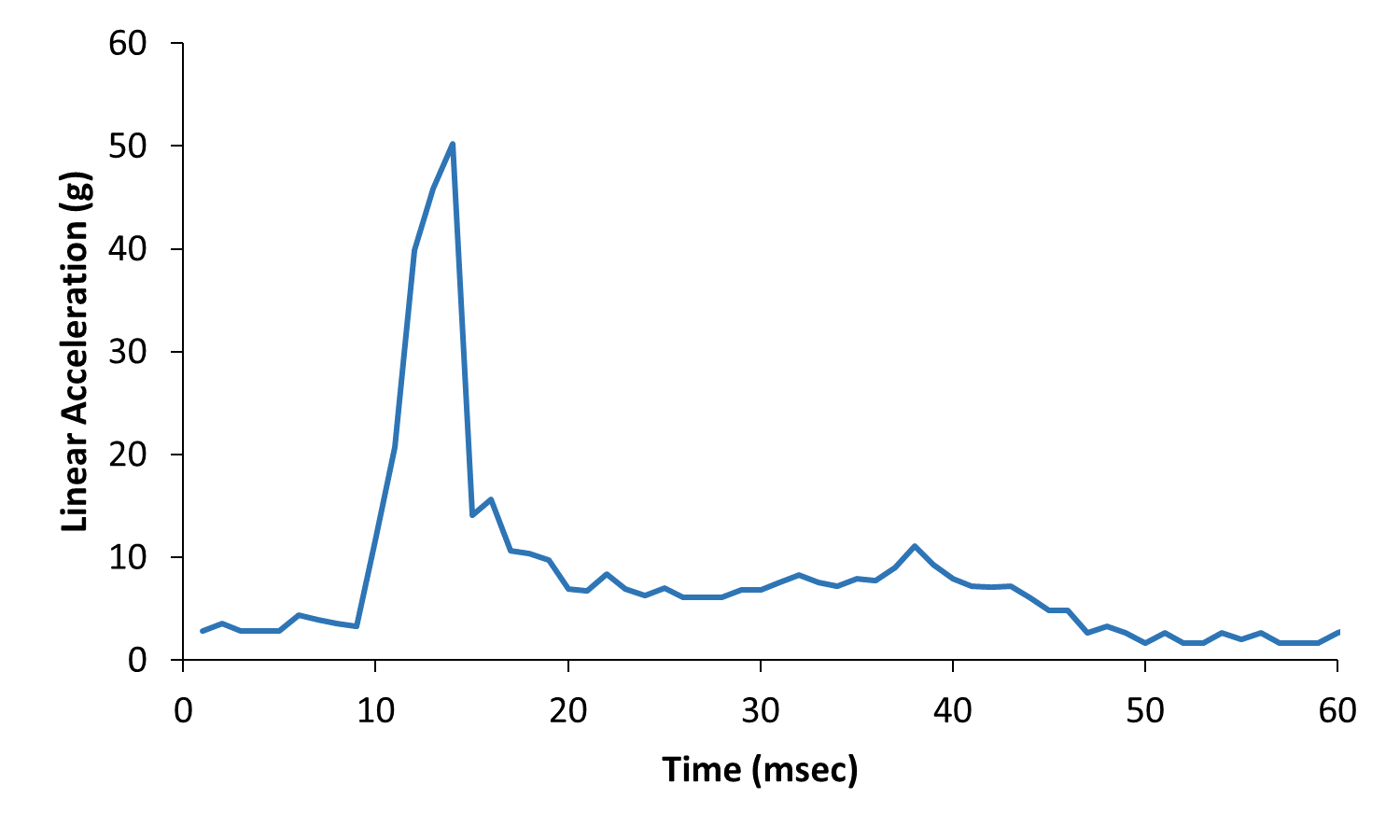 \| 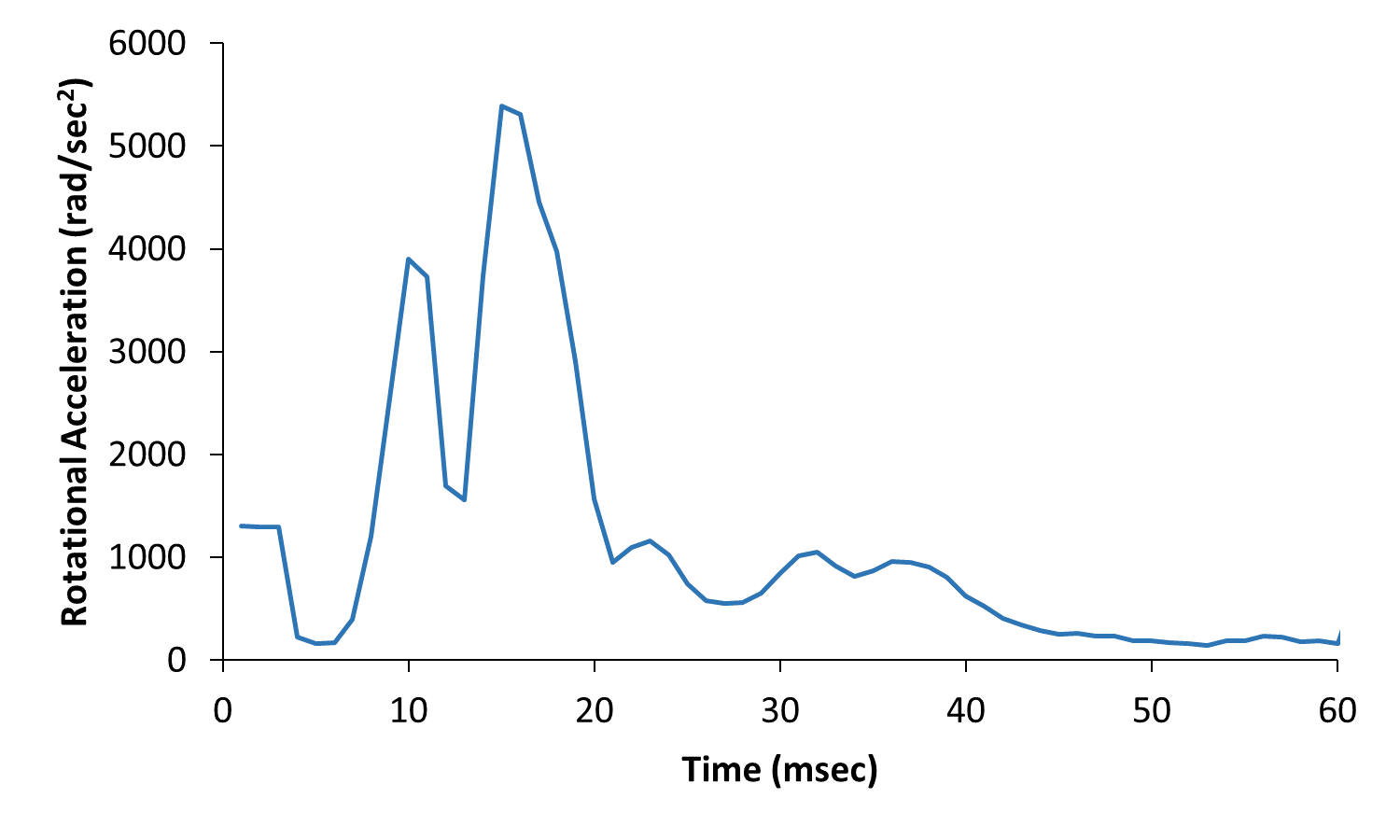 \| \| --- \| --- \|   Figure S2-1. Resultant linear and rotational acceleration time histories associated with a fall involving direct head impact onto the edge of a wooden bookcase. This fall generated the highest linear and rotational head accelerations.  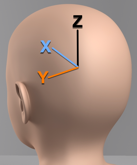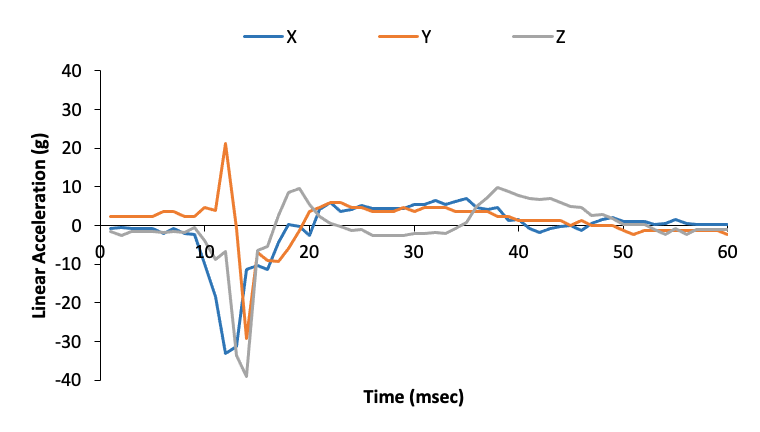 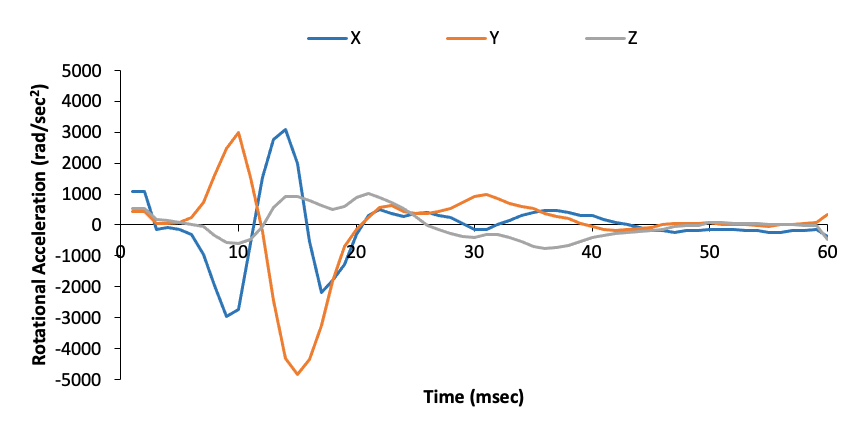  Figure S2-2. X, Y, and Z components of linear and rotational head acceleration time histories for Fall 1. \|  \| \| --- \| --- \| --- \| --- \| |
| --- | --- | --- | --- | --- |
| **Fall 2** - In another fall generating high levels of acceleration (31 g; 3812 rad/sec^2^; 12 msec impact duration; Figure S2-2), a child was climbing on the side of steps leading to a playground slide, positioned such that the majority of his weight was supported through his hands on the upper most step and through the heel of one foot positioned on the step below, while the other foot was freely suspended. While attempting to push himself upward, he lost his balance falling rearward to the ground (rubber mulch) impacting his buttocks, as his posterior torso and head impacted a metal support pole. The child cried immediately, touching his head while remaining in a seated position. A staff member picked up the child to comfort him; 17 sec after impact the child was released onto the playground and began engaging in play. No first aid was required, no medical attention was sought, and no injuries were reported.   \| 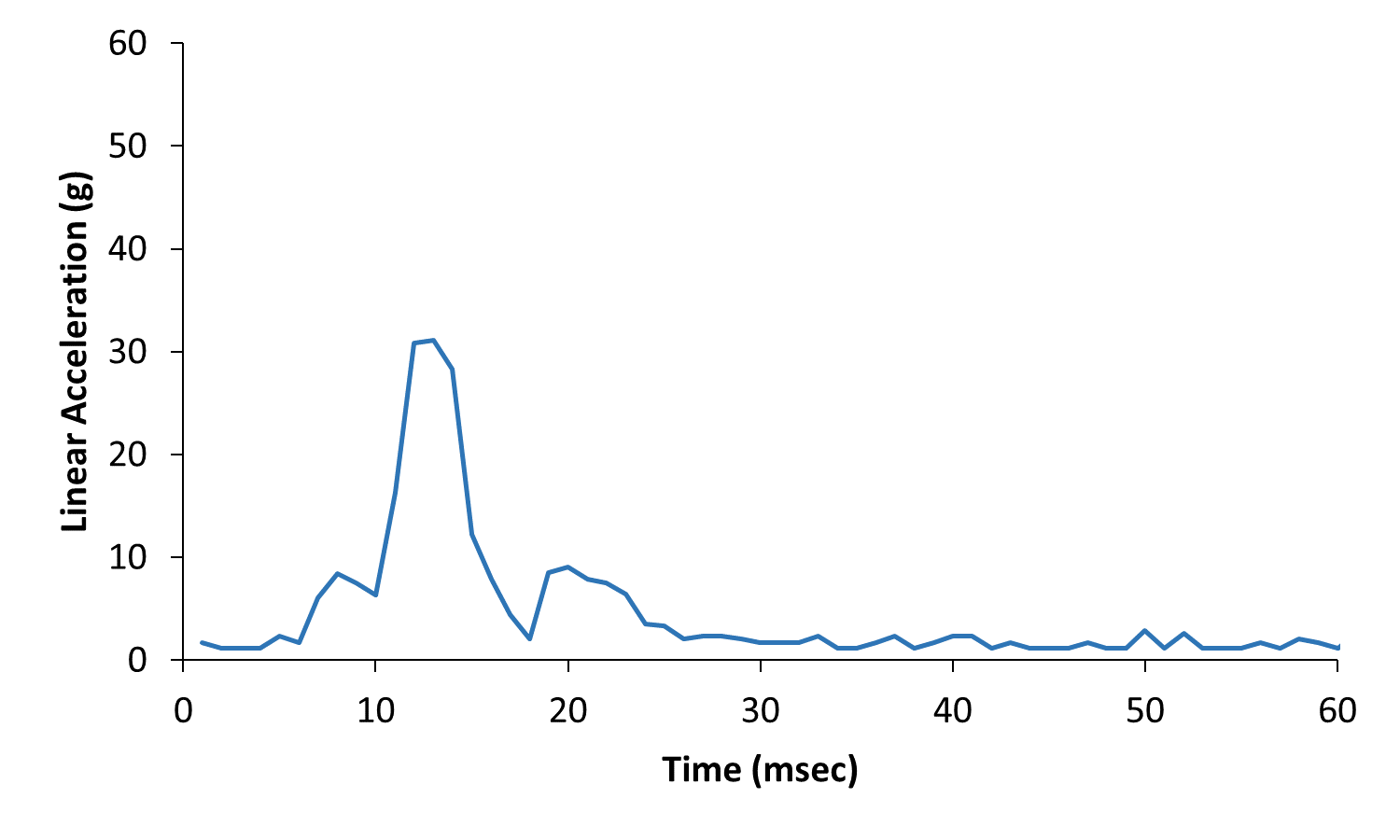 \| 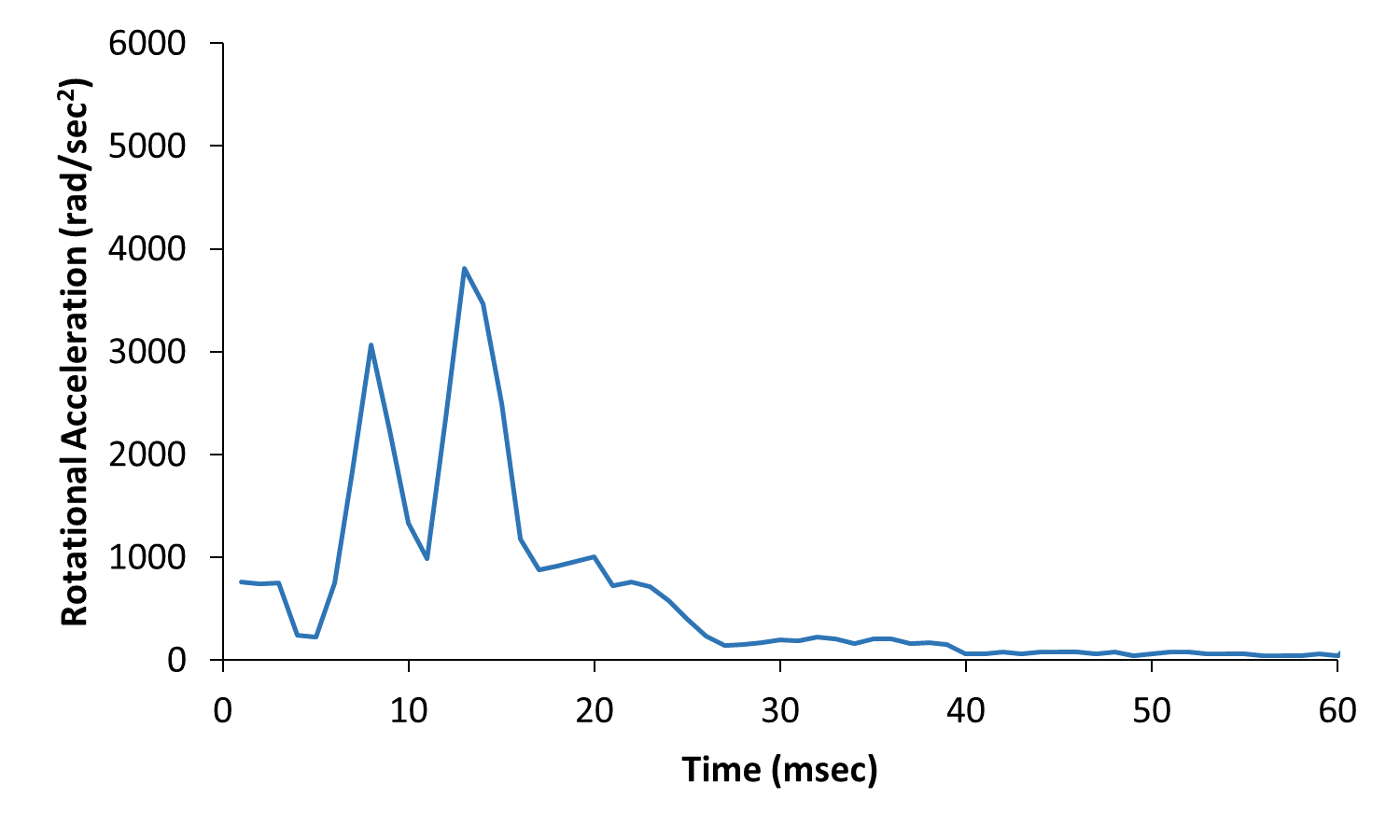 \| \| --- \| --- \|   Figure S2-3. Resultant linear and rotational acceleration time histories associated with a fall involving direct head impact onto a metal support pole.  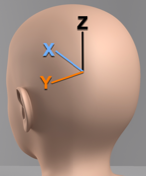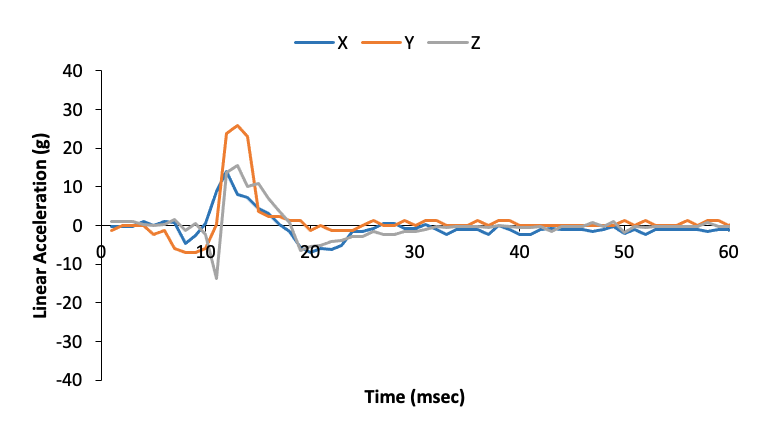 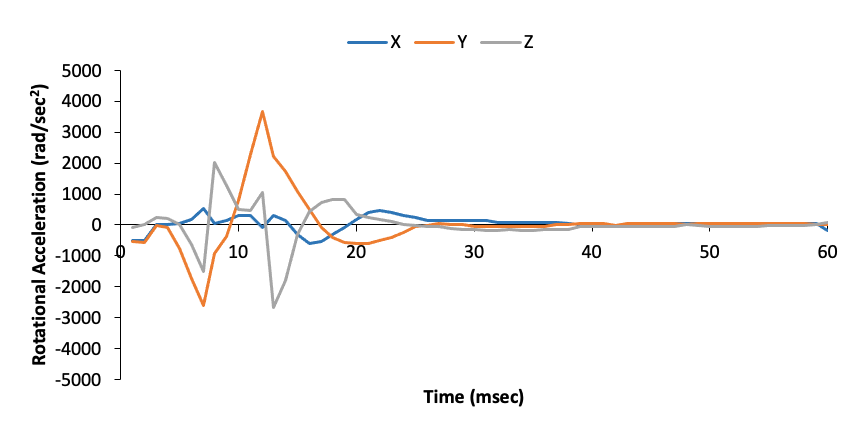  Figure S2-4. X, Y, and Z components of linear and rotational head acceleration time histories for Fall 2. |
| **Fall 3** - An event involving an adult falling onto a child also led to high levels of acceleration (38.3 g; 3625 rad/sec^2^; 16 msec impact duration; Figure S2-3). In this fall an adult childcare center staff member carrying a child (child A) stepped rearward inadvertently knocking over another child (child B) standing next to her, causing the staff member to fall onto child B and drop child A. Child B was wearing a SIM^TM^ G device, but child A was not enrolled in the study. As the adult stepped rearward, the lateral aspect of her leg bumped child B causing him to fall rearward impacting his buttocks, torso and then head on the linoleum floor ending in a supine position. Simultaneously the adult fell laterally onto child B impacting the lateral aspect of her pelvis onto the child’s upper torso and face, while concurrently dropping child A. As the adult fell onto child B, she attempted to support her weight on an outstretched hand and knee. Child B cried immediately and was picked up by another staff member and comforted. Approximately 6 minutes after the fall, child B was observed walking unassisted, although intermittently rubbing his head. 15 minutes after the fall child B was observed playing. No first aid was required, no medical care was sought, and no injuries were reported. (Child A had no apparent injuries as a result of the fall, but since he was not enrolled in the study, no follow-up was conducted.)   \| 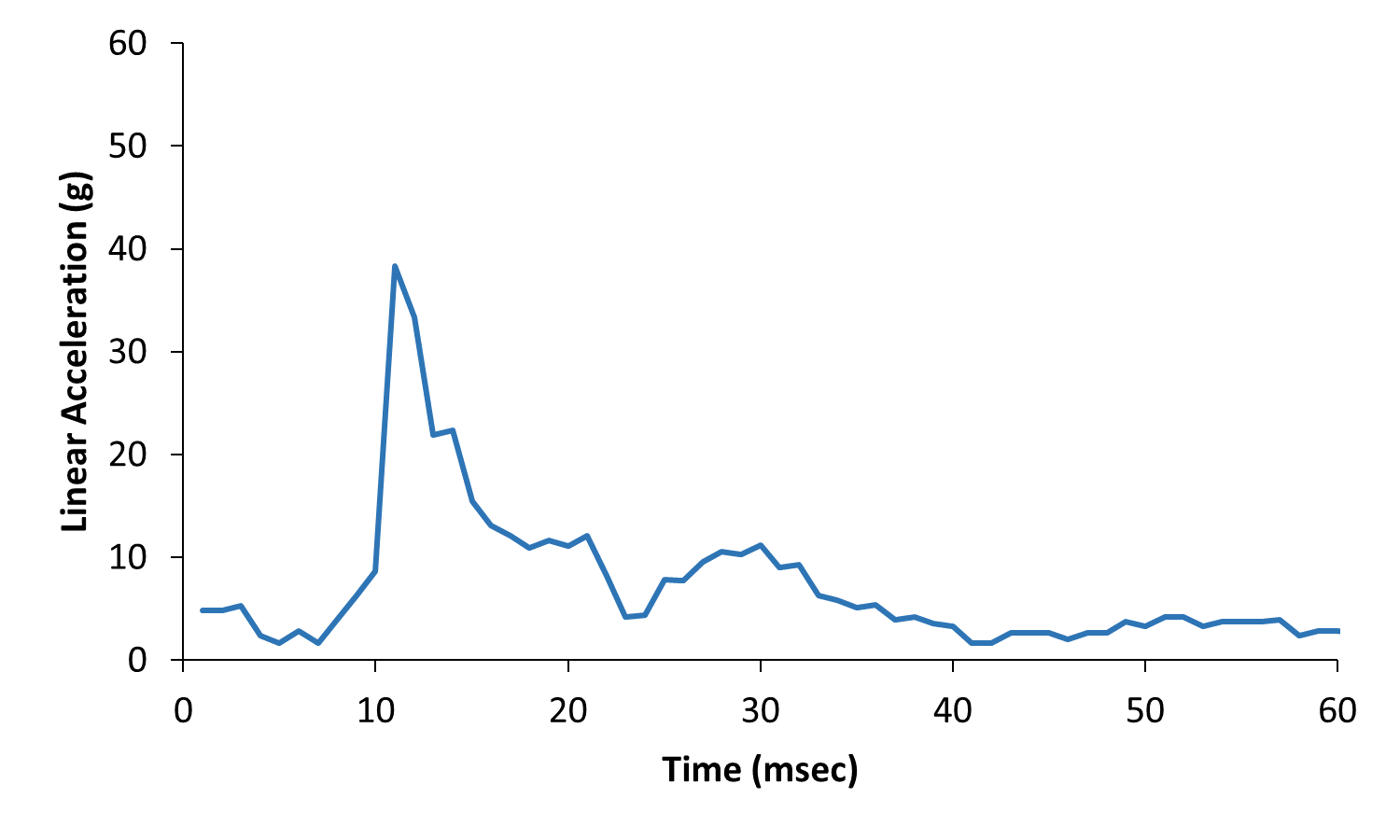 \| 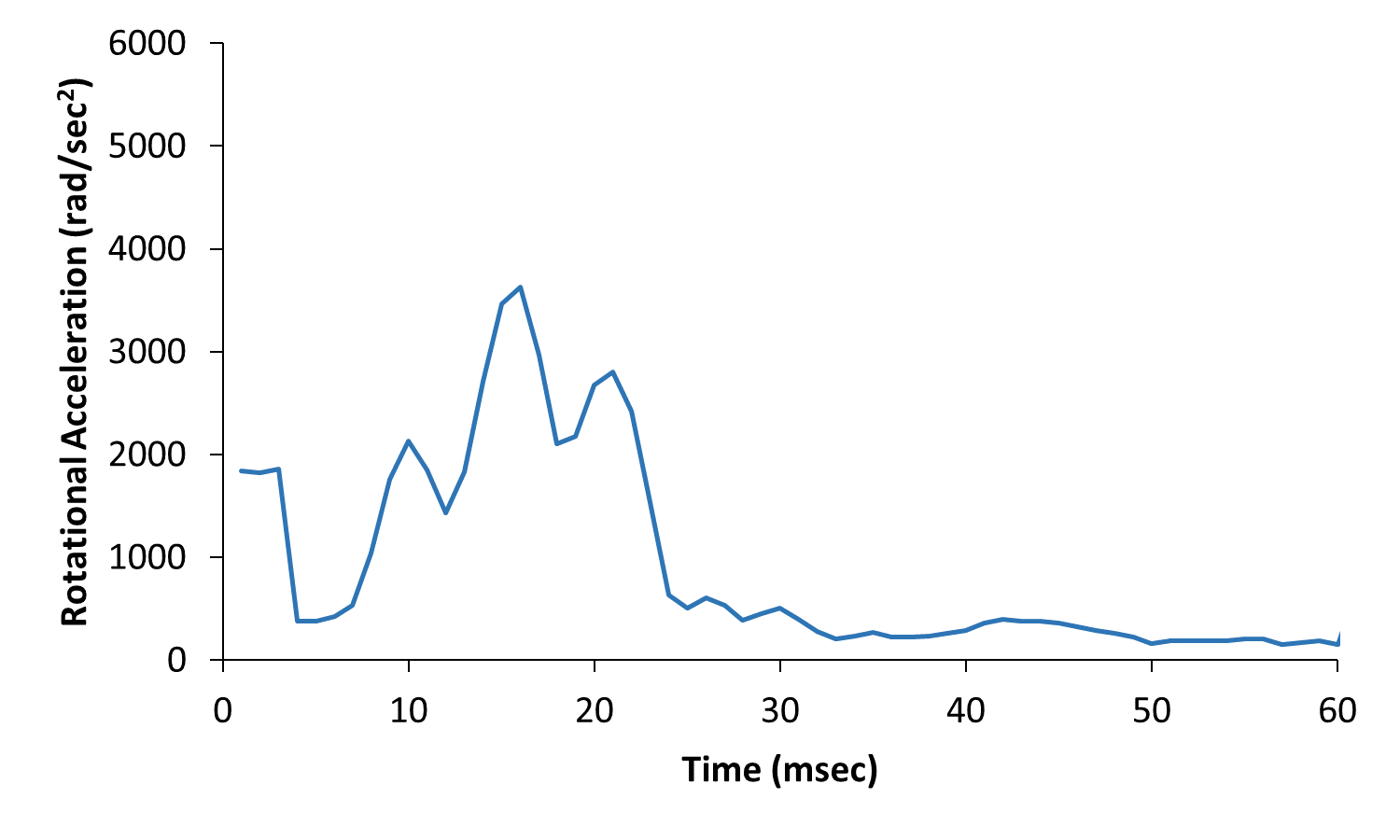 \| \| --- \| --- \|   Figure S2-5. Resultant linear and rotational acceleration time histories associated with a fall involving an adult falling onto a child.  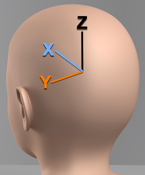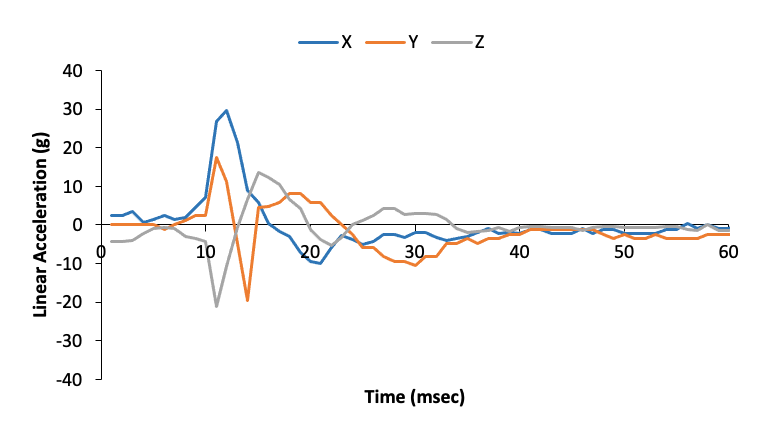 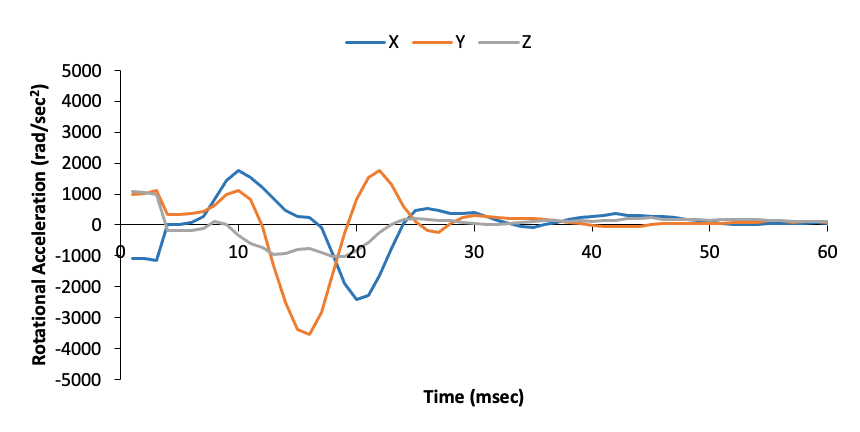  Figure S2-6. X, Y, and Z components of linear and rotational head acceleration time histories for Fall 3. |

**Supplementary Table S3. COR^a^ values measured in childcare environment**

| **Indoor Surfaces** | **COR Value** |  | **Outdoor and Other Surfaces** | **COR Value** |
| --- | --- | --- | --- | --- |
| Linoleum | 0.45 |  | Rubber playground mulch | 0.57 |
| Carpet | 0.41 |  | Rubber playground mushroom | 0.54 |
| Rug over carpet | 0.55 |  | Playground slide surface | 0.23 |
| Rug over linoleum | 0.45 |  | Playground slide edge | 0.48 |
| Foam pad | 0.47 |  | Playground slide platform | 0.34 |
| Carpeted stair | 0.45 |  | Playground slide steps | 0.18 |
| Drywall | 0.26 |  | Metal fence | 0.42 |
| Dense foam puzzle square | 0.61 |  | Foot of caterpillar tunnel | 0.66 |
| Foam furniture | 0.59 |  | Top of caterpillar tunnel | 0.36 |
| Wood furniture | 0.40 |  | Hard plastic playground tube | 0.26 |
| Plastic toy | 0.44 |  | Plastic panel of playground tube | 0.49 |
| Plastic storage bin top | 0.22 |  | Shoe (worn) | 0.38 |
| Plastic storage bin side | 0.36 |  | Soft tissue (arm) | 0.22 |
| Wicker basket bottom | 0.48 |  |  |  |
| Wicker basket side | 0.50 |  |  |  |
| Stuffed animal | 0.20 |  |  |  |
| Book | 0.39 |  |  |  |
| Folded blanket | 0.15 |  |  |  |
| Ball | 0.67 |  |  |  |

^a^ - COR was defined as (h/H)^0.5^, where H represents the initial height of a stainless-steel sphere dropped onto an impact surface and h represents the rebound height of the sphere as measured using a graduated glass tube that vertically constrains the sphere’s motion.
